# Supplementary material for: An Evaluation of Type 1 Interferon Related Genes in Male and Female-Matched, SARS-CoV-2 Infected Individuals Early in the COVID-19 Pandemic
Source: Viruses. 2024 Mar 20;16(3):472. doi: 10.3390/v16030472 (PMC10975322; doi:10.3390/v16030472)
Supplement: Supplementary file 1 [file viruses-16-00472-s001.zip › viruses-2778506-supplementary.pdf]

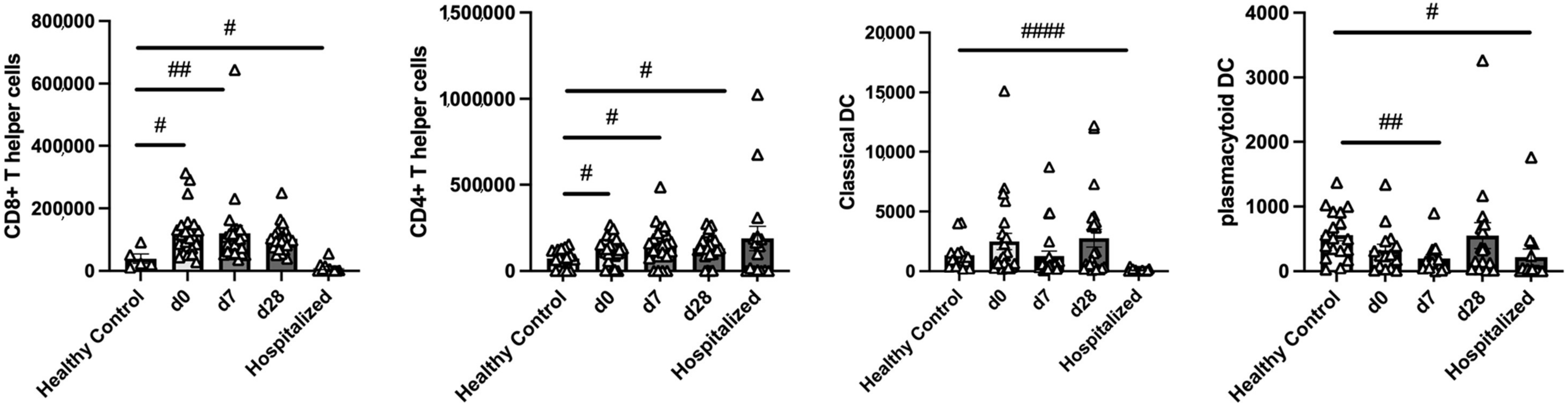

Supplementary Figure S1. Flow cyometric analysis of CD3+ CD8+ T cells, CD3+ CD4+ T cells, CD11c hi classical dendritic cells (DC), and CD11c + BDCA-1+ plasmacytoid DC in COVID-19+ individuals compared to healthy controls and hospitalized patients. Whole blood was collected from healthy controls and SARS-CoV2 infected (COVID-19+) people at three timepoints. Day 0 represents a blood draw on the day of SARS-CoV2 detection, day 7 is 1 week later, and day 28 is four weeks after SARS-COV2 detection. Hospitalized patients were all positive for SARS-CoV2 and blood was collected during their hospital stay. # indicates a significant difference ($p < 0.05$) by student t-test between indicated bars; ## $p < 0.01$, ### $p < 0.001$, #### $p < 0.0001$. Healthy controls, n =18, and for COVID-19+ individuals, n = 22.
